# Alice in Wonderland Syndrome as a Rare Presentation of Cryptogenic Stroke: A Case Report

**DOI:** 10.7759/cureus.79750

**Published:** 2025-02-27

**Authors:** Saba Ahmed, Samreen Ahmed, Jawad Zafar

**Affiliations:** 1 College of Medicine, Saint James School of Medicine, Arnos Vale, VCT; 2 Department of Behavioral Medicine and Psychiatry, Farhat Medical Clinic, Beckley, USA

**Keywords:** alice in wonderland syndrome (aiws), antipsychotics, auditory hallucinations, ischemic stroke, macropsia, neurological disorders, perceptual distortions, post-stroke symptoms, quetiapine treatment, visual hallucinations

## Abstract

Alice in Wonderland syndrome (AIWS) is a rare neurological disorder characterized by perceptual distortions, including alterations in body image perception and time perception. This case report presents a 94-year-old female patient who exhibited visual and auditory hallucinations, including macropsia (perception of objects as larger than their actual size), suggestive of AIWS, following an ischemic stroke. Despite initial suspicions of AIWS, imaging revealed chronic infarcts in the posterior right parietal lobe and the left occipital lobe, suggesting a stroke etiology. Management focused on symptomatic treatment with quetiapine, which effectively alleviated visual and auditory hallucinations, paranoia, and psychosis. This case explores the importance of thorough diagnostic evaluation to distinguish between post-stroke hallucinations versus those associated with AIWS. Additionally, it highlights the need for further research to expand on the mechanisms and optimal management strategies for AIWS, particularly in the context of stroke and other neurological conditions.

## Introduction

Alice in Wonderland syndrome (AIWS) encompasses a range of symptoms involving distortions in body image perception. This includes misperceptions of the sizes of body parts or external objects [1]. The condition was named after the main character in Lewis Carroll’s 1865 English children's novel "Alice's Adventures in Wonderland" by British psychiatrist John Todd in 1955. Just like in the book, AIWS encompasses the encounter of surreal sensations reminiscent of those experienced by the fictional character, including distorted body image, skewed size perception, and altered time perception. Symptoms can arise from migraines, temporal lobe epilepsy, brain tumors, the use of psychoactive drugs, and various infections [2].

This disorder is typically observed more frequently in young individuals compared to older adults [3]. While relatively overlooked for six decades, AIWS has recently garnered scientific interest owing to advancements in functional imaging methodologies [4]. This case report aims to explore the association between ischemic stroke in a patient and the symptoms that manifest as AIWS and to discuss potential management approaches for this presentation.

## Case presentation

A 94-year-old woman, with a medical history of type 2 diabetes, hypertension, hypothyroidism, gastroesophageal reflux disease (GERD), and anxiety, presented with the chief complaint, "I have been seeing things." The patient had recently been treated with 500 mg of ciprofloxacin twice daily for seven days for a urinary tract infection (UTI). She stated that she had been experiencing visual disturbances for the past year. She reported several visual hallucinations, including seeing multiple tall men walking in her backyard and room. She also described objects in her backyard as appearing larger than they actually were. During these episodes, she was unable to differentiate the hallucinations from reality. Additionally, she reported auditory hallucinations, stating that she constantly hears music and voices. She denied any paranoid delusions. Although her symptoms intensified during the recent UTI, they persisted in the absence of any infection or mood symptoms over the past year.

The patient underwent a thorough evaluation by a psychiatrist, including a complete neurological examination. No motor symptoms suggestive of Lewy body dementia or other neurological disorders associated with hallucinations were identified. Both dementia and delirium were ruled out at the time. The patient had been taking 25 mg of hydroxyzine, which she was instructed to discontinue due to its anticholinergic properties. She was prescribed 0.5 mg of risperidone to manage her visual distortions.

**Figure 1:**
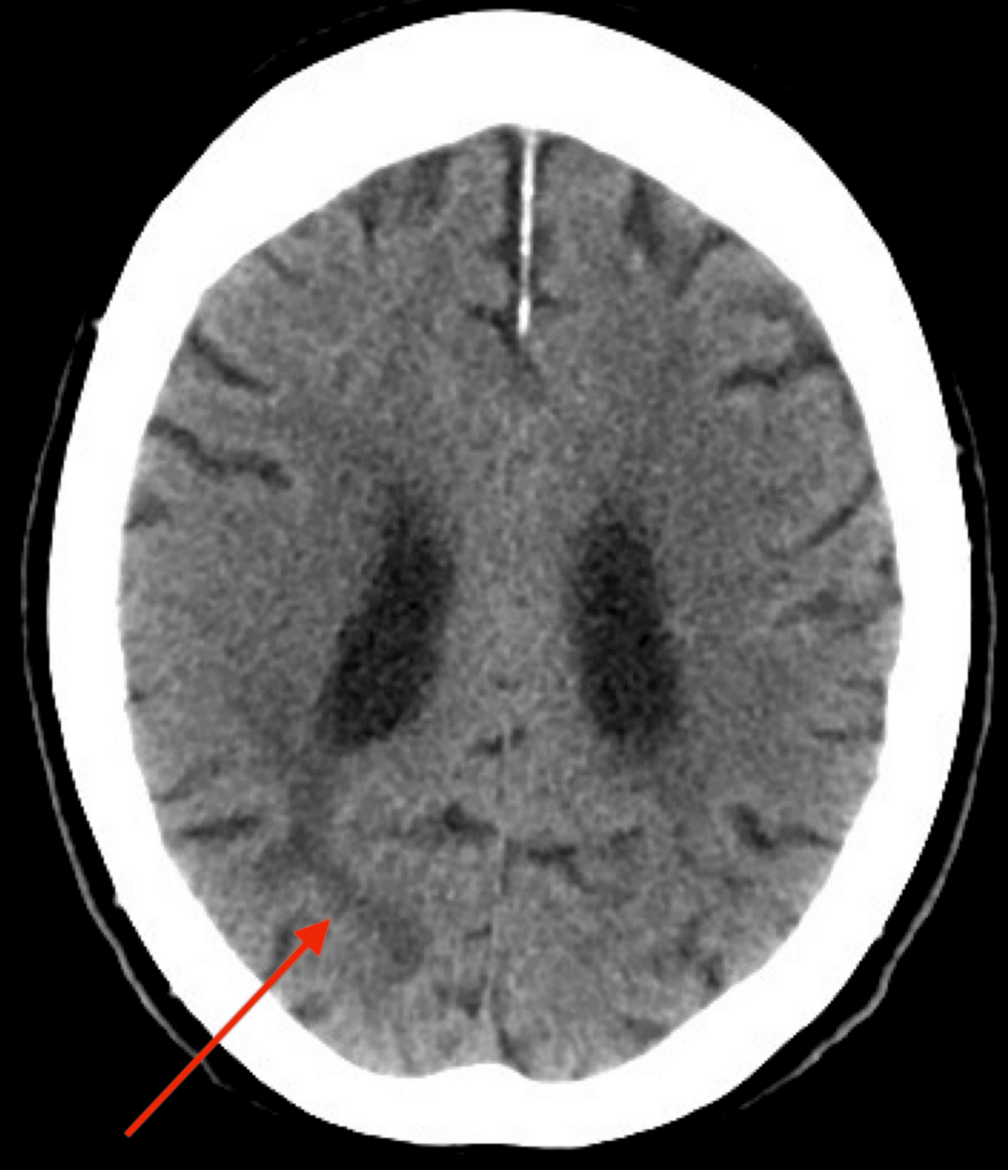
Right occipital lobe encephalomalacia Non-contrast axial CT scan of the brain showing an area of encephalomalacia in the right occipital lobe (red arrow), suggestive of a remote infarct.

**Figure 2:**
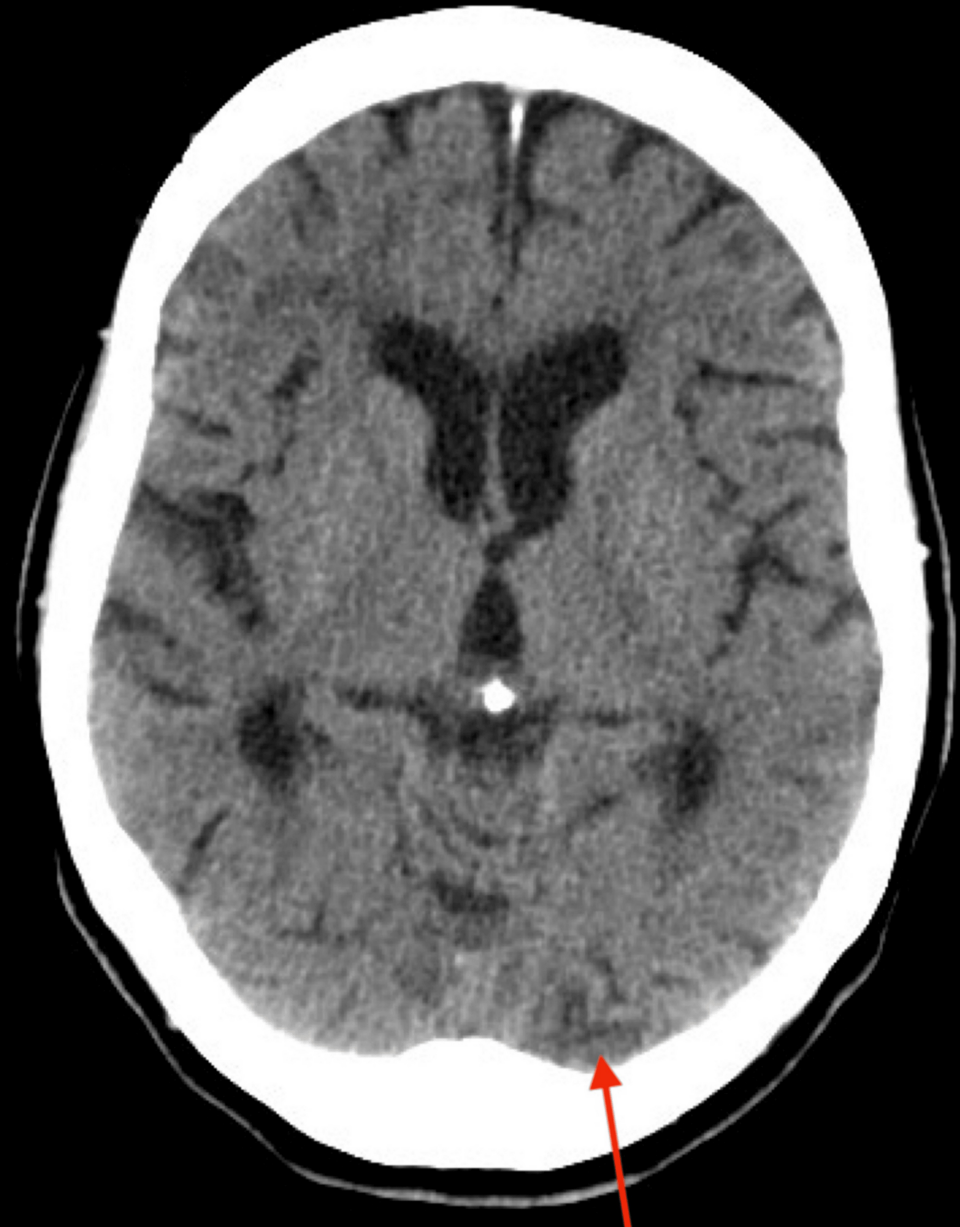
Left occipital lobe encephalomalacia Non-contrast axial CT scan of the brain demonstrating focal encephalomalacia in the left occipital lobe (red arrow), suggestive of a remote infarct.

**Figure 3:**
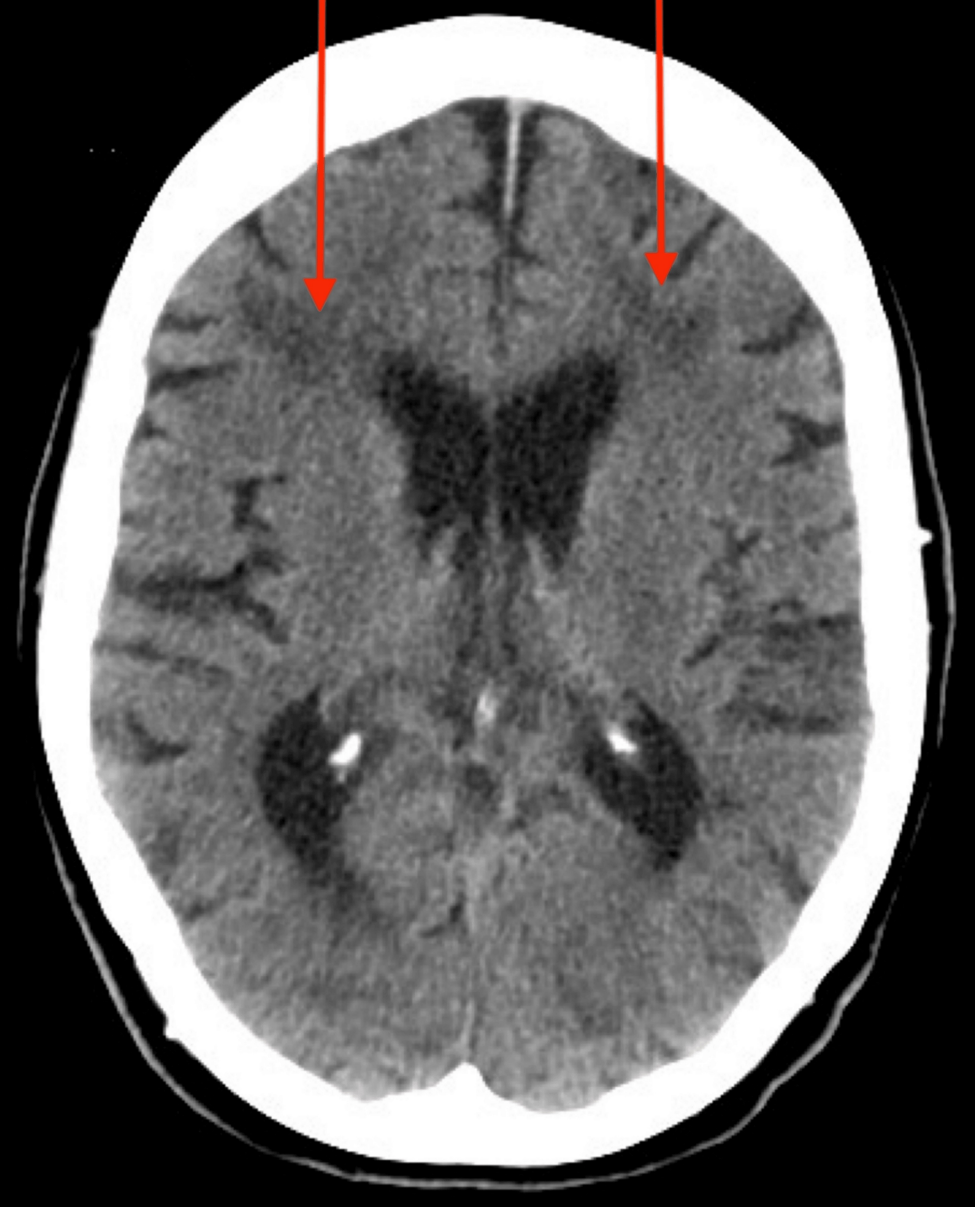
Microvascular ischemic changes Axial CT scan of the brain revealing mild, nonspecific low-attenuation white matter changes bilaterally (red arrows), likely representing chronic small vessel ischemic disease.

A CT scan was ordered. The scan revealed focal, small areas of encephalomalacia in the posterior right parietal lobe (Figure 1) and in the left occipital lobe (Figure 2), suggesting chronic infarcts. Additionally, mild nonspecific low-attenuation white matter changes, likely due to chronic small vessel ischemic disease, were observed (Figure 3). These findings indicate that the patient had a cryptogenic stroke. Due to an insurance-related issue, an MRI was not performed. 

After this initial visit, the patient was evaluated at another hospital for agitation, aggression, and confusion. She was told to discontinue 0.5 mg of risperidone and was instead prescribed 5 mg of olanzapine. The patient returned to the clinic seven days later and reported continued visual hallucinations, significant auditory hallucinations, poor sleep, and paranoia. She was advised to discontinue olanzapine due to its lack of improvement in her symptoms and was instead prescribed 25 mg of quetiapine in the morning and 50 mg of quetiapine at bedtime.

Nine days later, the patient returned and reported feeling better, with improvements in visual and auditory hallucinations, as well as sleep. She was advised to continue taking 50 mg of quetiapine twice a day, as it effectively managed her symptoms of paranoia and psychosis.

## Discussion

The patient in this case presented with both auditory and visual hallucinations, but what set her symptoms apart was her perception of objects appearing larger than their actual size. This experience, termed macropsia, could point to a diagnosis of AIWS, a condition characterized by distortions in visual perception, body image, and time perception [4].

Research into 166 documented cases of AIWS identified migraine as the most common cause (27.1%), followed by infections, with the highest number of cases coming from Epstein-Barr virus (22.9%). Other contributing factors encompass brain lesions, medication/drug usage, psychiatric disorders, epilepsy, and peripheral nervous system diseases [5]. Initially, the patient's symptoms aligned with those typically associated with AIWS. However, contrary to expectations of migraine or infection, a CT scan revealed chronic infarcts in the posterior right parietal lobe and the left occipital lobe, suggesting a cryptogenic stroke.

It is crucial to differentiate between visual hallucinations post-stroke and those associated with AIWS. The symptoms, in this case, are more likely to correspond to AIWS rather than post-stroke visual hallucinations because, in post-stroke hallucinations, individuals perceive nonexistent objects, whereas, in AIWS, real objects appear distorted [3].

AIWS in post-stroke patients is not a completely unknown phenomenon. This can be seen in the following cases. One case involved a 95-year-old woman who reported distortions, such as perceiving individuals with tiny hands and family members with enlarged heads. Imaging revealed a hypodensity in the territory of the right posterior cerebral artery, leading to a diagnosis of cryptogenic ischemic stroke [6]. Another case involved a 45-year-old man who experienced persistent belief in the enlargement of his left upper limb during rehabilitation, with symptoms triggered by stressful situations post stroke [7]. Another paper cited two cases, involving symptoms of micropsia and macropsia, linked to lesions in the right occipital lobe caused by isolated cortical venous thrombosis (ICVT). One of the patients received lacosamide therapy and experienced one year without recurrence, suggesting a possible association between cortical venous thrombosis, epilepsy, and AIWS symptoms [3].

Treatment focused on managing underlying symptoms, with quetiapine proving effective. While most AIWS cases are benign, underlying causes may necessitate symptomatic treatment, with antiepileptics commonly used [6]. Research suggests that antipsychotics are infrequently used, and their efficacy is typically viewed as limited. Additionally, when distortions occur alongside psychotic symptoms, it is important to recognize that antipsychotics, such as risperidone, may occasionally trigger or worsen these distortions by lowering the threshold for epileptic activity [4]. In this current case, it was sufficient for the patient to stay on an antipsychotic and remain symptom-free.

## Conclusions

This case highlights a rare presentation of AIWS following an ischemic stroke in an elderly patient. While AIWS is typically associated with migraines, infections, or epilepsy, this case underscores the importance of considering stroke as a potential cause, particularly when visual and perceptual distortions such as macropsia are present. A thorough diagnostic evaluation is critical to distinguishing post-stroke hallucinations from AIWS. The patient's symptoms were effectively managed with quetiapine, demonstrating its potential in treating AIWS-associated psychosis and hallucinations. This case adds to the limited body of knowledge on AIWS in post-stroke patients and emphasizes the need for further research to clarify the mechanisms and develop optimal treatment approaches for AIWS in the context of stroke and other neurological disorders.
